# Screening of Fungi for Antimycobacterial Activity Using a Medium-Throughput Bioluminescence-Based Assay

**DOI:** 10.3389/fmicb.2021.739995

**Published:** 2021-09-06

**Authors:** Alexander B. J. Grey, Melissa M. Cadelis, Yiwei Diao, Duckchul Park, Thomas Lumley, Bevan S. Weir, Brent R. Copp, Siouxsie Wiles

**Affiliations:** ^1^Bioluminescent Superbugs Lab, Department of Molecular Medicine and Pathology, School of Medical Sciences, The University of Auckland – Waipapa Taumata Rau, Auckland, New Zealand; ^2^School of Chemical Sciences, The University of Auckland – Waipapa Taumata Rau, Auckland, New Zealand; ^3^Manaaki Whenua – Landcare Research, Auckland, New Zealand; ^4^Department of Statistics, The University of Auckland – Waipapa Taumata Rau, Auckland, New Zealand

**Keywords:** mycobacteria, *Mycobacterium marinum*, *Mycobacterium abscessus*, bioluminescence, luciferase, minimum inhibitory concentration, screening, antibacterial

## Abstract

There is a real and urgent need for new antibiotics able to kill Mycobacteria, acid-fast bacilli capable of causing multiple deadly diseases. These include members of the *Mycobacterium tuberculosis* complex, which causes the lung disease tuberculosis (TB) as well as non-tuberculous Mycobacteria (NTM) a growing cause of lung, skin, soft tissue, and other infections. Here we describe a medium-throughput bioluminescence-based pipeline to screen fungi for activity against Mycobacteria using the NTM species *Mycobacterium abscessus* and *Mycobacterium marinum.* We used this pipeline to screen 36 diverse fungal isolates from the International Collection of Microorganisms from Plants (ICMP) grown on a wide variety of nutrient-rich and nutrient-poor media and discovered that almost all the tested isolates produced considerable anti-mycobacterial activity. Our data also provides strong statistical evidence for the impact of growth media on antibacterial activity. Chemical extraction and fractionation of a subset of the ICMP isolates revealed that much of the activity we observed may be due to the production of the known anti-mycobacterial compound linoleic acid. However, we have identified several ICMP isolates that retained their anti-mycobacterial activity in non-linoleic acid containing fractions. These include isolates of *Lophodermium culmigenum*, *Pseudaegerita viridis*, and *Trametes coccinea*, as well as an unknown species of *Boeremia* and an isolate of an unknown genus and species in the family *Phanerochaetaceae*. Investigations are ongoing to identify the sources of their anti-mycobacterial activity and to determine whether any may be due to the production of novel bioactive compounds.

## Introduction

There is a real and urgent need for new antibiotics able to kill Mycobacteria, acid-fast bacilli capable of causing multiple deadly diseases. Because of their slow growth and hydrophobic, lipid-rich outer membrane, treatment of mycobacterial infections can take months to years and require multiple antibiotics (Seaworth and Griffith, 2017; Pontali et al., 2019). The major mycobacterial human pathogens are members of the *Mycobacterium tuberculosis* complex, which causes the lung disease tuberculosis (TB) (Gagneux, 2018), described by the World Health Organization as a global epidemic. Also of concern are the non-tuberculous Mycobacteria (NTM), free living opportunistic pathogens that are ubiquitous in the environment and able to cause lung, skin, and soft tissue infections (Mirsaeidi et al., 2014; Gonzalez-Santiago and Drage, 2015; Koh and Schlossberg, 2017). Almost two hundred NTM species have been identified to date, which were recently found to divide into five clades based on phylogenetic characteristics (Gupta et al., 2018). Rates of NTM infections are increasing globally, including in hospital settings (Al-Mahruqi et al., 2009; Roux et al., 2009; Moore et al., 2010; Morimoto et al., 2014; Donohue and Wymer, 2016; Donohue, 2018; Ratnatunga et al., 2020). NTM are natural inhabitants of water and their inclusion in implanted devices such as catheters, prosthetics, and pacemakers, have resulted in cases of bacteremia and disseminated infection, while NTM outbreaks have been associated with invasive procedures such as cosmetic surgeries, intramuscular injections, and tattooing (Griffin et al., 2019; Jabbour et al., 2020). Recently, some cases of pulmonary infections with *Mycobacterium chimaera* were traced back to site of manufacture of heater-cooler units routinely used during open heart surgery (Williamson et al., 2017).

Aotearoa New Zealand is an archipelago which split from the Gondwanan supercontinent approximately 85 million years ago and has since gradually become more isolated from other land masses (Wallis and Trewick, 2009). This geographical separation has led to the evolution of iconic native flora, fauna, and fungi. The Crown Research Institute Manaaki Whenua is the custodian of the International Collection of Microorganisms from Plants (ICMP) (Johnston et al., 2017). The ICMP contains over 10,000 fungal cultures derived from plants and soil from Aotearoa New Zealand and the South Pacific. The collection has a great diversity of fungal species, host substrates, and collection localities, with the earliest cultures dating from the 1960s. While the collection contains some of the fungal genera traditionally used for antibiotic production it has not been rigorously tested for antimicrobial activity against mycobacterial species. In our view, this makes the ICMP an excellent and untapped resource for antibiotic discovery.

The search for new antibiotics with activity against Mycobacteria is complicated by their slow growth, with species like *M. tuberculosis* having a doubling time of approximately 24 h. Mycobacteria also tend to clump in liquid culture due to their hydrophobic cell envelope. These properties make the two most common methods of measuring antibacterial activity, the production of zones of inhibition when grown on agar, or degree of turbidity when grown in liquid culture, slow and unreliable. Tagging bacteria with the genes that encode for luciferase-based reporters allows light to be used as a rapid surrogate marker for bacterial viability (Andreu et al., 2012). We and others have shown that bioluminescence is an excellent non-destructive real-time reporter to assay for anti-mycobacterial activity in microtiter plate formats using a luminometer (Andreu et al., 2012; Dalton et al., 2016; Early et al., 2019; Chengalroyen et al., 2020; Jain et al., 2020) or *in vivo* using sensitive imaging equipment (Andreu et al., 2013).

Here we describe a medium-throughput bioluminescence-based pipeline to screen fungi for activity against Mycobacteria using the NTM species *Mycobacterium abscessus* and *Mycobacterium marinum.* Our results indicate that many of the ICMP fungal isolates are anti-mycobacterial and have identified isolates of *Lophodermium culmigenum*, *Pseudaegerita viridis*, and *Trametes coccinea*, as well as an unknown species of *Boeremia* and an isolate of an unknown genus and species in the family *Phanerochaetaceae* as suitable for further study.

## Materials and Methods

### Bacterial Strains and Growth Conditions

In this study, we used *M. abscessus* BSG301 (Cadelis et al., 2021) and *M. marinum* BSG101 (Dalton et al., 2017) which are stable bioluminescent derivatives transformed with the integrating plasmid pMV306G13ABCDE (Andreu et al., 2010). We grew mycobacterial cultures with shaking (200 rpm) in Middlebrook 7H9 broth (Fort Richard, New Zealand) supplemented with 10% Middlebrook ADC enrichment media (Fort Richard, New Zealand), 0.4% glycerol (Sigma-Aldrich, New Zealand) and 0.05% tyloxapol (Sigma-Aldrich, New Zealand). We grew *M. abscessus* at 37°C and *M. marinum* at 28°C.

### Fungal Material

Fungal isolates (Table 1) were provided by Manaaki Whenua – Landcare Research, a New Zealand Crown Research Institute responsible for the curation of the International Collection of Microorganisms from Plants (ICMP). We stored fungal isolates individually in cryotubes at −80°C. We made freezer stocks by growing each fungus on 1.5% Potato Dextrose Agar (PDA) and excising small cubes of agar (5–6 mm in length) from the fungus’ growing edge. We placed these cubes within a cryovial containing 1 mL of 10% glycerol and rested them for 1 h after which we removed the remaining liquid glycerol and stored the tubes at -80°C.

**TABLE 1:** Fungal isolates used in this study.

Fungus	ICMP number	GenBank Accession	Description
*Agaricales* sp.	17554	MT107903	An undescribed crust fungus in the cyphellaceae – a family closely related to mushroom species but forming crust or simple hood-like fruitbodies. It was isolated in Kerikeri, New Zealand in August 2007
*Aleurodiscus* sp.	16336	MZ325955	*Aleurodiscus* sp. is a pinkish crust fungus. This culture was isolated from dead wood near Lake Waikaremoana, New Zealand in May 1985
*Amylostereum sacratum*	10158	MZ325952	*Amylostereum sacratum* is a plant pathogen causing root rot. This culture was isolated from an apple tree in Nelson, New Zealand in May 1977
*Aspergillus terreus*	477	MW862777	*Aspergillus terreus* is a common cosmopolitan saprotrophic soil-inhabiting fungus. This culture was isolated in September 1961 in Auckland, New Zealand from sheep’s wool incubated at 30°C
*Boeremia* sp.	17650	MW862790	This isolate is an unknown species of *Boeremia* which are often plant pathogens. It was isolated from the surface of a mushroom in the Mamaku Plateau, New Zealand in May 1991
*Cerrena zonata*	16347	MW862786	*Cerrena zonata* is a white rot decay fungus of dead wood. This culture was isolated from Ngāruawāhia, New Zealand in April 1995
*Chalara scabrida*	20449	MK432752	*Chalara scabrida* is an endemic saprobic fungus. The culture was isolated from a living *Phormium cookianum* leaf in Mt Hutt, New Zealand in February 2014
*Cunninghamella echinulata*	1083	MZ325951	*Cunninghamella echinulata* is a common soil saprotroph. This culture was isolated from Auckland, New Zealand in December 1978
*Cylindrobasidium* sp.	16397	MZ325956	This isolate is a crust fungus in the family physlacriaceae and related to the *Armillaria* mushroom. This culture was isolated from apple wood in Auckland, New Zealand in June 1973
*Dentipellis leptodon*	18110	MZ325966	*Dentipellis leptodon* grows on the underside of dead wood and has dangling spines and is related to the Lion’s Mane *Hericium* fungus. This culture was isolated from *Metrosideros robusta* wood in Mamaku, New Zealand in March 1984
*Helicodendron triglitziense*	16004	MK432688	*Helicodendron triglitziense* is an aero-aquatic species isolated from dead alder leaves in Horseshoe Lake Reserve wetland, Christchurch, New Zealand in June 2005
*Hyaloscypha spinulosa*	16865	MK432695	*Hyaloscypha spinulosa* is an aero-aquatic species isolated from a dead rimu twig in Pigeon Bay, New Zealand in September 2006
*Hypholoma australianum*	21474	MZ325972	*Hypholoma australianum* is an orange mushroom with a white stem. This culture was isolated from wood buried in soil in Otago Lakes, New Zealand in May 2016
*Laetiporus portentosus*	15555	MZ325953	*Laetiporus portentosus* is a soft bracket fungus, traditionally used as a tinder and wound packing material by Mâori, the indigenous people of New Zealand. This culture was isolated from a beech tree in Rimutaka Forest Park, New Zealand in May 1999
*Lanzia allantospora*	15649	AY755334	*Lanzia allantospora* is an endemic cup fungus found on kauri wood in Northland, New Zealand in April 1992
*Lauriomyces bellulus*	15050	EF029218	*Lauriomyces bellulus* is a saprophytic fungus. This culture was isolated from a dead leaf of *Weinmannia racemosa* in Katikati, New Zealand in May 2003
*Lentinellus pulvinulus*	16586	MW862787	*Lentinellus pulvinulus* is a white rot wood decay mushroom. This culture was isolated from a dead wood in Pehitawa Kahikatea Forest Reserve, New Zealand in May 2006.
*Lentinula novae-zelandiae*	18003	MZ325965	*Lentinula novae-zelandiae* is a native edible “shitake” mushroom. This culture was isolated from dead wood in Dunedin, New Zealand in September 1991
*Linnemannia elongate*	17447	MZ325962	*Linnemannia elongate* is a Mucorales fungus. The culture was isolated from a kauri tree in Rotorua, New Zealand in January 2008
*Lophodermium culmigenum*	18328	MZ325968	*Lophodermium culmigenum* is a plant decay fungus. This culture was isolated from Trounson Kauri Park, Chatham Islands, New Zealand in November 1992
*Metapochonia bulbillosa*	18174	MZ325967	*Metapochonia bulbillosa* is an insect pathogen fungus. This culture was isolated from dead leaves of Marram grass in Lake Tennant, New Zealand in 1985
*Mortierella* sp.	20597	MZ325970	This isolate is an unknown species of *Mortierella*, a common Mucorales soil fungus. This culture was isolated from rotting wood from Farewell Spit, New Zealand in May 2014
*Mucor laxorrhizus*	20877	MZ325971	*Mucor laxorrhizus* is a Mucorales saprobe. This culture was isolated from rotten wood from a stream in St Arnaud, New Zealand in January 2015
*Neodidymelliopsis* sp.	11463	MW862783	This isolate is an unknown species of *Neodidymelliopsis* which are typically plant pathogens. This culture was isolated from *Pittosporum* leaves in Albany, Auckland, New Zealand in October 1991
*Peniophora lycii*	16714	MZ325959	*Peniophora lycii* is a crust fungus. This culture was isolated from decaying wood in Te Waiiti, New Zealand in May 2001
*Phanerochaetaceae* sp.	18785	MZ325969	This isolate is an unknown genus and species of crust fungi in the family *Phanerochaetaceae.* The culture was isolated from beech leaves from Matakitaki, New Zealand in December 2010
*Pleurotus australis*	18149	MH395972	*Pleurotus australis* is an edible wood decay mushroom. This culture was isolated from the Waitakere Ranges near Auckland, New Zealand in February 1987
*Pleurotus purpureo-olivaceus*	9630	MH395959	*Pleurotus purpureo-olivaceus* is an edible wood decay mushroom. This culture was isolated from Manapouri, New Zealand in May 1990
*Pleurotus purpureo-olivaceus*	17077	GQ411512	*Pleurotus purpureo-olivaceus* is an edible wood decay mushroom. This culture was isolated from the Craigieburn Range, New Zealand in May 2006
*Pseudaegerita viridis*	16864	MZ325960	*Pseudaegerita viridis* is an aero-aquatic species. This culture was isolated from a dead rimu twig in Pigeon Bay, New Zealand in September 2006
*Stereum* sp.	16953	MZ325961	This isolate is an unknown species of *Stereum*, a wood decay bracket fungus, and was isolated from Rangitoto Station, New Zealand in November 2006
*Torrendiella brevisetosa*	18823	JN225946	*Torrendiella brevisetosa* is a cup fungus. This culture was isolated from beech leaves in Matakitaki, New Zealand in December 2010
*Trametes coccinea*	13182	MW862784	*Trametes coccinea* is a wood decay bracket fungus. This culture was isolated from a dead radiata pine in Northland, New Zealand in September 1985
*Umbelopsis* sp.	17492	EU770239	This isolate is an unknown species of *Umbelopsis*, a Mucorales saprobe, isolated from grapevines in Whenuapai, New Zealand in April 2007
*Vararia fusispora*	17544	MZ325963	*Vararia fusispora* is a crust fungus. This culture was isolated from a decaying rimu branch in Owhango, New Zealand in October 2007
*Xylariaceae* sp.	16006	MZ325954	This isolate is an unknown genus and species of the *Xylariaceae* family that was isolated from Ahuriri Reserve, Christchurch, New Zealand in May 2005

### Fungal DNA Extraction and ITS Sequencing

We used a small portion of mycelium from growing fungi and extracted DNA using the REDExtract-N-Amp^TM^ Plant PCR Kit (Sigma-Aldrich) according to the manufacturer’s protocol. We diluted DNA samples five-fold and amplified using the ITS1F (5′ CTTGGTCATTTAGAGGAAGTAA 3′) and ITS4 (5′ TCCTCCGCTTATTGATATGC 3′) primer set in a 10 μl reaction volume using the REDExtract-N-Amp Plant PCR Kit (Sigma-Aldrich) according to the manufacturer’s instructions. We used the following PCR conditions: initial denaturation at 94°C for 3 min, followed by 40 cycles of denaturation at 94°C for 30 s, annealing at 52°C for 30 s and extending at 72°C for 30 s. The final extension was performed at 72°C for 7 min. We checked the amplified DNA by gel electrophoresis before sequencing using an Applied Biosystems^TM^ 3500xL Genetic Analyzer using both ITS1F and ITS4 primers. We trimmed and combined the sequence data using Geneious (Geneious Biologics), removed any low-quality reads and used BLAST to check fungal identification. Optimized sequence data were aligned using MEGA7 (Kumar et al., 2016).

### Primary Fungal Screening

We grew fungal isolates on PDA (Fort Richard, New Zealand) prior to screening for antibacterial activity using a 24 well plate assay. Briefly, we added 0.5 mL aliquots of agar to triplicate wells of a black 24 well plate (4titude, Millennium Science, New Zealand) and allowed them to set. We obtained all media from Fort Richard (New Zealand). In addition to PDA, these comprised: Czapek Solution Agar (CSA), Czapek Yeast Extract Agar (CYA), Malt Extract Agar (MEA), Malt Yeast Extract Agar (MYA), Oatmeal Agar (OA), Rice Extract Agar (REA), and Tryptone Yeast Extract Agar (TYA). With the aid of a sterile scalpel blade, we sectioned fungal isolates grown on PDA into cubes ≤5 mm in diameter, and then transferred the cubes to the agar-filled wells of the 24-well plates ensuring that each cube was placed fungus-side down and touching the agar. We covered the inoculated 24-well screening plates, sealed them with parafilm, and incubated them at room temperature.

We monitored fungal growth visually at regular intervals and recorded the time taken for them to either cover the entire well or to stop visibly growing. At twice this time, we removed a 6 mm plug of agar from each well using a biopsy punch. To screen for antibacterial activity, we resuspended *M. abscessus* BSG301 and *M. marinum* BSG101 in 0.8% Middlebrook 7H9 broth (Fort Richard, New Zealand) supplemented with 10% Middlebrook ADC enrichment media (Fort Richard, New Zealand) to a final concentration of 10^7^ colony forming units (CFU)/mL for *M. abscessus* and 10^8^ CFU/mL for *M. marinum*. With the aid of a pipette, we pipetted 50 μL of the bacterial-agar mixture into the cylindrical holes left after removal of the fungal-agar plugs and allowed the mixture to set. We measured bacterial luminescence at regular intervals using a Victor X-3 luminescence plate reader (PerkinElmer) with an integration time of 1 s. Between measurements, plates were covered, placed in a plastic box lined with damp paper towels, and incubated static at 37°C for *M. abscessus* and 28°C for *M. marinum*. We performed these assays three times. We have published a more detailed description of our methods on the protocol repository website protocols.io (Wiles and Grey, 2021a,b).

### Fungal Fermentation and Extraction

We grew fungal cultures either in liquid media or on solid media at room temperature and then freeze-dried them. We extracted the dry cultures with MeOH (Sigma-Aldrich, New Zealand) for 4 h followed by CH_2_Cl_2_ (Sigma-Aldrich, New Zealand) overnight. We concentrated the combined organic extracts under reduced pressure and subjected the crude extracts to C_8_ reversed-phase column chromatography eluting with a gradient of H_2_O/MeOH (Sigma-Aldrich, New Zealand) to afford five fractions (F1–F5). Full details are provided in Supplementary Material.

### Extract Screening

We grew mycobacterial cultures until they reached stationary phase (approximately 3–5 days for *M. abscessus* BSG301 and 7–10 days for *M. marinum* BSG101) and then diluted these in Mueller Hinton broth II (MHB) (Fort Richard) supplemented with 10% Middlebrook ADC enrichment media and 0.05% tyloxapol to give an optical density at 600 nm (OD_600_) of 0.001 which is the equivalent of ∼10^6^ bacteria per mL. We dissolved the fungal fractions in DMSO (Sigma-Aldrich, New Zealand) and added these in duplicate to the wells of a black 96-well plate (Nunc, Thermo Scientific) at doubling dilutions with a maximum concentration of 50 mg/mL. Then we added 50 μL of diluted bacterial culture to each well of the fraction containing plates giving final extract concentrations of 0–1000 μg/mL and a cell density of ∼5 × 10^5^ CFU/mL.

We used the antibiotic rifampicin (Sigma-Aldrich, New Zealand) as a positive control at 1000 μg/mL for *M. abscessus* and 10 μg/mL for *M. marinum*. Between measurements, plates were covered, placed in a plastic box lined with damp paper towels, and incubated with shaking at 100 rpm at 37°C for *M. abscessus* and 28°C for *M. marinum*. We measured bacterial luminescence at regular intervals using a Victor X-3 luminescence plate reader (PerkinElmer) with an integration time of 1 s. We have defined the MIC as causing a 1 log reduction in light production, as previously described (Dalton et al., 2016, 2017). We have published a more detailed description of our methods on the protocol repository website protocols.io (Wiles and Grey, 2021c,d).

### General Chemistry Conditions

We recorded NMR spectra using a Bruker Avance DRX-400 spectrometer or an Avance III-HD 500 spectrometer operating at 400 MHz or 500 MHz for ^1^H nuclei and 100 MHz or 125 MHz for ^13^C nuclei utilizing standard pulse sequences at 298 K. We recorded high resolution mass spectra on a Bruker micrOTOF QII (Bruker Daltonics, Bremen, Germany). We carried out analytical thin layer chromatography (TLC) on 0.2 mm thick plates of DC-plastikfolien Kieselgel 60 F254 (Merck). We carried out reversed-phase column chromatography on C_8_ support with a pore size of 40–63 μm (Merck). We carried out gel filtration chromatography on Sephadex LH-20 (Pharmacia). We carried out flash chromatography on Diol-bonded silica with a pore size of 40–63 micron (Merck). We used solvents that were of analytical grade or better and/or purified according to standard procedures.

### Statistical Analysis

We fitted a logistic mixed model for activity with a random effect for biologic replicates. We tested the main effects and second-order interactions of the variables, using the lme4 and car packages in R (Bates et al., 2015; Fox and Weisberg, 2019; R Core Team, 2020).

## Results

### Identification of Novel Fungal Taxa Endemic to Aotearoa New Zealand

**FIGURE 1:**
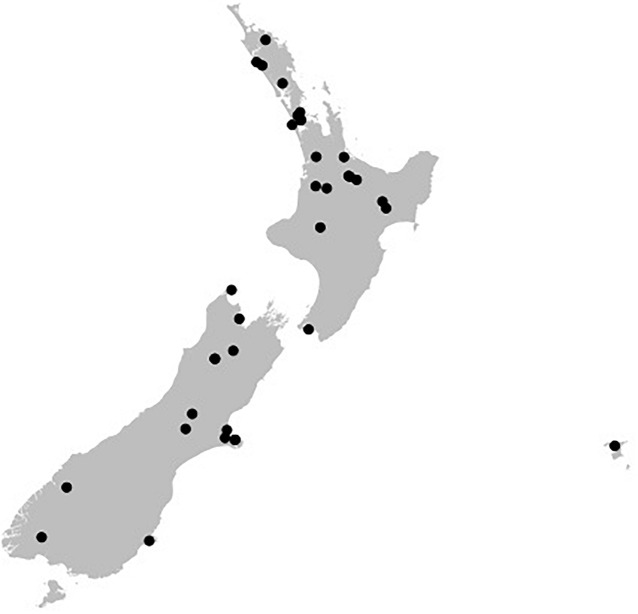
Geographical spread of the isolation locations for the fungal isolated used in this study within the archipelago of Aotearoa New Zealand. Black dots are individual ICMP isolates.

The 36 ICMP fungal isolates used in this study were collected between 1961 and 2016 and from locations across Aotearoa New Zealand, including the North, South, and Chatham Islands (Figure 1). Of the 36 isolates, nine were not able to be identified as a known species and one is from both an unknown genus and species (Table 2). These isolates likely represent novel taxa endemic to Aotearoa New Zealand. As with the broader collection, they cover a range of isolation dates, the earliest being isolated in 1973 (*Cylindrobasidium* sp. ICMP 16397) and the most recent in 2014 (*Mortierella* sp. ICMP 20597). They also cover a broad range of isolation locations within New Zealand, from Kerikeri in the North Island (*Agaricales* sp. ICMP 17554) to Christchurch in the South Island (*Xylariaceae* sp. ICMP 16006).

**TABLE 2:** ICMP isolates belonging to novel fungal taxa likely endemic to Aotearoa New Zealand.

Phylum	Fungus	ICMP number	GenBank Accession	Isolation substrate and location	Isolation year
Ascomycota	*Boeremia* sp.	17650	MW862790	Isolated from the surface of a mushroom in the Mamaku Plateau	1991
	*Neodidymelliopsis* sp.	11463	MW862783	Isolated from *Pittosporum* leaves in Albany, Auckland	1991
	*Xylariaceae* sp.	16006	MZ325954	Isolated in Ahuriri Reserve, Christchurch	2005
Basidiomycota	*Agaricales* sp.	17554	MT107903	Isolated in Kerikeri, Northland	2007
	*Aleurodiscus* sp.	16336	MZ325955	Isolated from dead wood near Lake Waikaremoana	1985
	*Cylindrobasidium* sp.	16397	MZ325956	Isolated from apple wood in Auckland	1973
	*Phanerochaetaceae* sp.	18785	MZ325969	Isolated from beech leaves from Matakitaki	2010
	*Stereum* sp.	16953	MZ325961	Isolated at Rangitoto Station	2006
Mucoromycota	*Mortierella sp.*	20597	MZ325970	Isolated from rotting wood from Farewell Spit	2014
	*Umbelopsis* sp.	17492	EU770239	Isolated from grapevines in Whenuapai	2007

### Whole Cell Screening Identified Many ICMP Fungal Isolates as Having Anti-mycobacterial Activity

**FIGURE 2:**
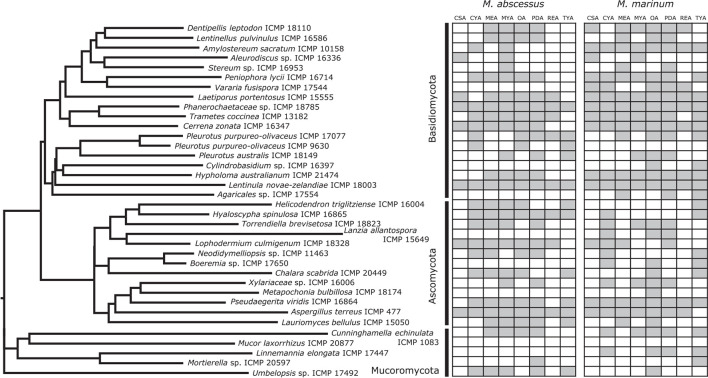
Phylogeny and activity of ICMP isolates grown in different media against *M. abscessus* BSG301 and *M. marinum* BSG101. Active media for each isolate are shown in gray. CSA, Czapek Solution Agar; CYA, Czapek Yeast extract Agar; MEA, Malt Extract Agar; MYA, Malt Yeast extract Agar; OA, Oatmeal Agar; PDA, Potato Dextrose Agar; REA, Rice Extract Agar; TYA, Tryptone Yeast extract Agar. ICMP isolates are described as active if they caused a minimum 1-log (90%) reduction in the bioluminescence of the mycobacterial strains. The phylogenetic tree was constructed by comparing ITS sequences to those in GenBank and the phylogenetic tree was constructed using the Molecular Evolutionary Genetics Analysis version 7.0 software (MEGA7) using the neighbor-joining method and p-distance as the substitution model. Each phylogeny was tested using the bootstrap method with 500 replications.

**FIGURE 3:**
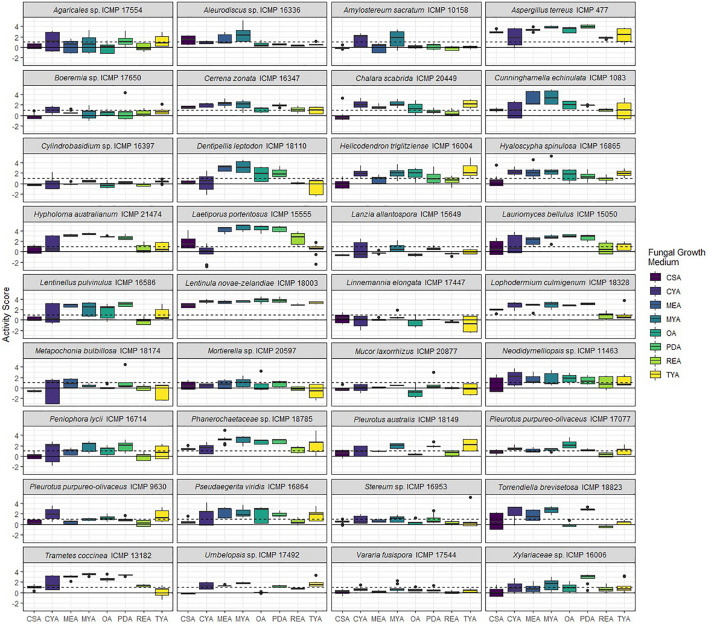
Antibacterial activity of ICMP fungal isolates against *Mycobacterium abscessus* BSG301 when grown on different media. Data is presented as box and whisker plots of activity scores. The solid line shown at 0 is the median control value while the dotted line at 1 is the activity threshold. Scores above 1 correspond to a >90% reduction in bacterial bioluminescence compared to the corresponding no-fungi control. Similarly, an activity score above 2 means corresponds to a >99% reduction. CSA, Czapek Solution Agar; CYA, Czapek Yeast Extract Agar; MEA, Malt Extract Agar; MYA, Malt Yeast Extract Agar; OA, Oatmeal Agar; PDA, Potato Dextrose Agar; REA, Rice Extract Agar; TYA, Tryptone Yeast Extract Agar. Boxes are upper and lower quartiles with median shown. The whiskers extend up to 1.5× the inter-quartile range and any dots beyond those bounds are outliers.

**FIGURE 4:**
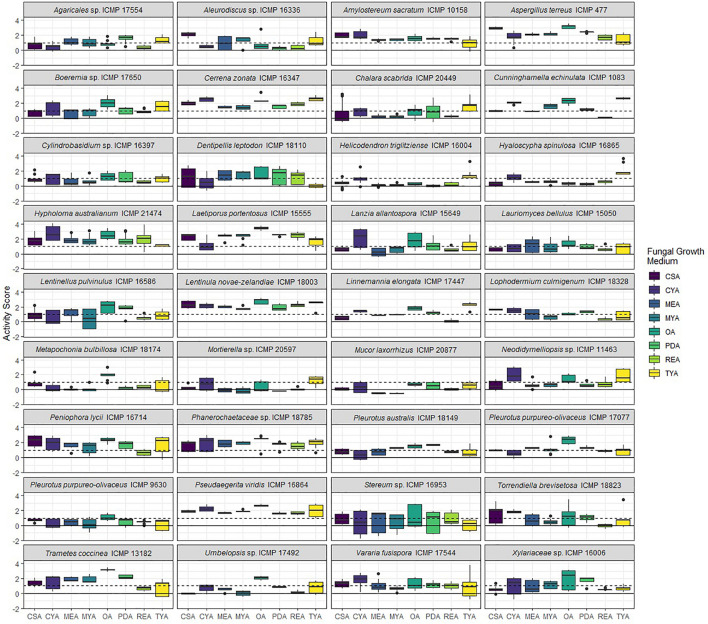
Antibacterial activity of ICMP fungal isolates against *Mycobacterium marinum* BSG101 when grown on different media. Data is presented as box and whisker plots of activity scores. The solid line shown at 0 is the median control value while the dotted line at 1 is the activity threshold. Scores above 1 correspond to a >90% reduction in bacterial bioluminescence compared to the corresponding no-fungi control. Similarly, an activity score above 2 means corresponds to a >99% reduction. CSA, Czapek Solution Agar; CYA, Czapek Yeast Extract Agar; MEA, Malt Extract Agar; MYA, Malt Yeast Extract Agar; OA, Oatmeal Agar; PDA, Potato Dextrose Agar; REA, Rice Extract Agar; TYA, Tryptone Yeast Extract Agar. Boxes are upper and lower quartiles with median shown. The whiskers extend up to 1.5× the inter-quartile range and any dots beyond those bounds are outliers.

We screened 36 ICMP fungal isolates for antibacterial activity against *M. abscessus* BSG301 and *M. marinum* BSG101. The isolates belong to three different fungal Phyla, and we grew them on eight different media giving us a total of 288 fungi-media combinations tested for each bacterium (Figures 2–4).

We measured antibacterial activity as reductions in light output of our bioluminescent mycobacterial strains over a 72-h period. We calculated activity scores by first converting the luminescence measurement at each time-point into an area under the curve (AUC) value for each well. We then divided this number by the median AUC of a sterile control plate inoculated and incubated at the same time as the fungus-containing plates. The negative log of this value corresponds to the activity score. We define a fungus-media combination as active/antibacterial if the median activity score is above 1 which corresponds to a > 90% reduction in light compared to the control. Similarly, an activity score above 2 means corresponds to a > 99% reduction.

We observed no consistent difference in activity by mycobacterial strain, but there is strong statistical evidence of differences in activity between media and fungal Phyla, and that these vary by mycobacterial strain (Table 3).

**TABLE 3:** Analysis of Deviance Table (Type II Wald Chi^2^ tests).

	Chi^2^	Degrees of freedom	Significance
Fungal phyla	2.57	2	*p* = 0.28
Mycobacterial strain	3.48	1	*p* = 0.06
Fungal growth medium	273.30	7	*p* < 0.0001
Fungal Phyla × Mycobacterial strain	99.50	2	*p* < 0.0001
Fungal Phyla × fungal growth medium	117.96	14	*p* < 0.0001
Mycobacterial strain × fungal growth medium	124.95	7	*p* < 0.0001

#### More ICMP Fungal Isolates Are Active Against *M. marinum* Than *M. abscessus*

We observed that 28/36 (77%) fungal isolates were active against *M. abscessus* when grown in at least one medium, with 130/288 (45%) fungi-medium combinations being anti-mycobacterial against this bacterium (Figures 2, 3). In contrast, 34/36 (94%) fungal isolates were active against *M. marinum*, with 146/288 (51%) fungi-medium combinations being anti-mycobacterial against this bacterium (Figures 2, 4). Of the two fungal isolates that were not active against *M. marinum* when grown in any of the media tested, only the Mucoromycota fungus *Mucor laxorrhizus* ICMP 20877 displayed no activity against *M. abscessus*. The second isolate, *Pleurotus purpureo-olivaceus* ICMP 9630 was active against *M. abscessus* when grown on CYA, MYA, and TYA. However, a second isolate of *P. purpureo-olivaceus* we tested, ICMP 17077, was active against both mycobacterial strains. We could discern no obvious pattern between fungal species or genus for those isolates that were only active against *M. marinum*, namely *Agaricales* sp. ICMP 17554, *Boeremia* sp. ICMP 17650, *Cylindrobasidium* sp. ICMP 16397, *Lanzia allantospora* ICMP 15649, *Linnemannia elongata* ICMP 17447, *Metapochonia bulbillosa* ICMP 18174, and *Vararia fusispora* ICMP 17544.

The fungal isolates we tested belong to three Phyla: the Basidiomycota (18 ICMP isolates), the Ascomycota (13 ICMP isolates), and the Mucoromycota (5 ICMP isolates) (Figure 2). We observed that the group with the most active fungi-medium combinations was the Basidiomycota (55%), followed by the Mucoromycota (52%), and then the Ascomycota (46%%) (Figure 2). More Basidiomycota-medium combinations were active against *M. marinum* [92/144 (64%)] (Figures 2, 4) than *M. abscessus* [67/144 (47%)] (Figures 2, 3). In contrast, more Ascomycota-medium combinations were active against *M. abscessus* [53/104 (51%)] (Figures 2, 3) than *M. marinum* [43/104 (41%)] (Figures 2, 4).

#### Differential Impact of Growth Medium on Anti-mycobacterial Activity

We observed that many of the ICMP fungi displayed differential activity depending on their growth medium with the majority being active on more than one medium. An isolate of the native New Zealand “shiitake” mushroom *Lentinula novae-zelandiae* (ICMP 18003) and an isolate of unknown genus and species in the family *Phanerochaetaceae* (ICMP 18785) were active against both *M. abscessus* and *M. marinum* when grown on an all 8 media (Figures 2–4). An isolate of *Aspergillus terreus* (ICMP 477) was also active against *M. abscessus* regardless of growth media (Figures 2, 4), while *Amylostereum sacratum* ICMP 10158, *Cerrena zonata* ICMP 16347, *Hypholoma australianum* ICMP 21474, *Laetiporus portentosus* ICMP 15555, and *Pseudaegerita viridis* ICMP 16864 were active against *M. marinum* when grown on all media (Figures 2, 4).

When assessing for activity against *M. abscessus*, 2/36 fungal isolates were only active when grown on one of the eight media, an unknown species of *Mortierella* (ICMP 20597) when grown on PDA and an unknown species of *Stereum* (ICMP 16953) on MYA (Figures 2, 3). ICMP 20597 was also only active against *M. marinum* when grown on one of the eight media, though in this case it was TYA (Figures 2, 4). Three other fungal isolates were only active against *M. marinum* when grown on one of the eight media, *Helicodendron triglitziense* ICMP 16004 on TYA, and *Metapochonia bulbillosa* ICMP 18174 and an unknown species of *Umbelopsis* (ICMP 17492) on OA (Figures 2, 4).

#### Potato Dextrose Agar (PDA) Is the Most Active Culture Medium for Screening for Anti-mycobacterial Activit*y*

We observed that PDA was the most active culture medium with 29/36 (80%) fungal isolates active against either bacterium (Figure 2). For *M. marinum*, the most active culture medium was OA, followed by PDA (24 and 23 active fungi, respectively) while for *M. abscessus* it was MYA followed by PDA (24 and 22 active fungi, respectively). We observed that isolates grown on REA and CSA were the least active, with only 14/36 isolates (39%) being active against either mycobacterium species when cultured on these media (Figure 2). MYA and MEA were the two media that favored *M. abscessus* activity, with 24 fungal isolates active when grown on MYA and 20 isolates active when grown on MEA, compared to 17 and 16 being active against *M. marinum*, respectively (Figure 2).

### Screening of Extracts and Fractions From ICMP Fungal Isolates for Anti-mycobacterial Activity

We prepared extracts from 41 fungus-medium combinations which were further separated into 5 fractions, designated F1–F5. Fraction F1 (100% water) is generally comprised of sugars while fraction F5 (100% methanol) contains predominantly fatty acids and sterols. Fractions F2, F3, and F4 typically contain the chemical compounds we are most interested in pursuing, with the potential to be bioactive.

**FIGURE 5:**
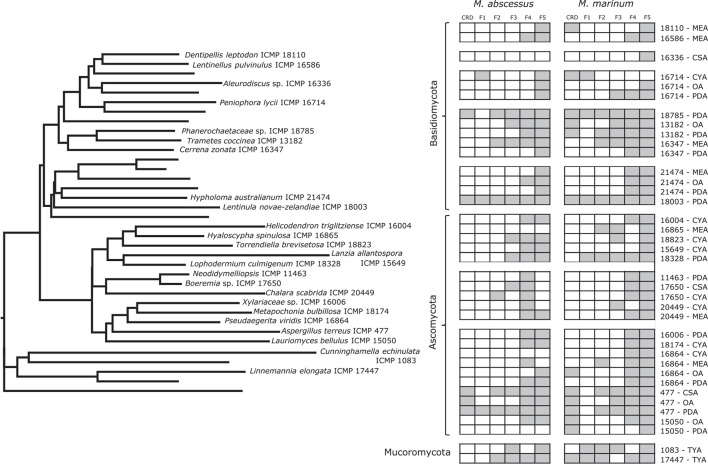
Phylogeny and activity of crude extracts and fractions from ICMP isolates grown in different media against *M. abscessus* BSG301 and *M. marinum* BSG101. Active extracts and fractions for each isolate are shown in gray. CSA, Czapek Solution Agar; CYA, Czapek Yeast extract Agar; MEA, Malt Extract Agar; MYA, Malt Yeast extract Agar; OA, Oatmeal Agar; PDA, Potato Dextrose Agar; REA, Rice Extract Agar; TYA, Tryptone Yeast extract Agar. ICMP isolates are described as active if they caused a minimum 1-log (90%) reduction in the bioluminescence of the mycobacterial strains. The phylogenetic tree was constructed by comparing ITS sequences to those in GenBank and the phylogenetic tree was constructed using the Molecular Evolutionary Genetics Analysis version 7.0 software (MEGA7) using the neighbor-joining method and p-distance as the substitution model. Each phylogeny was tested using the bootstrap method with 500 replications.

**FIGURE 6:**
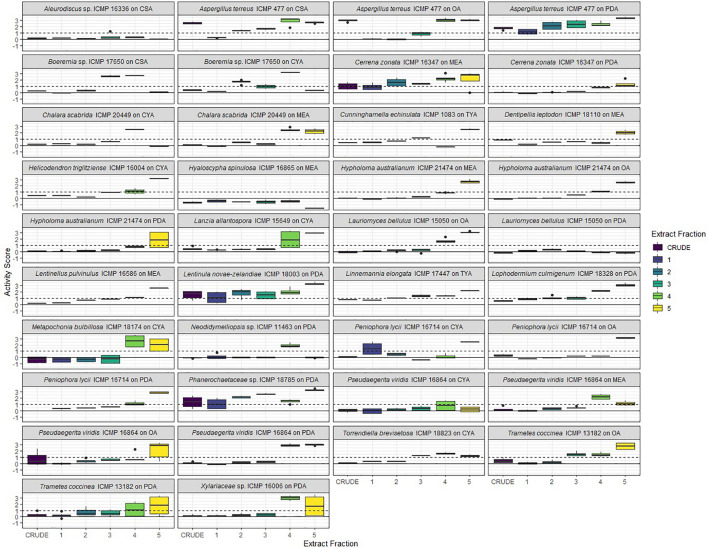
Antibacterial activity of crude extracts and fractions 1–5 from ICMP fungal isolates against *Mycobacterium marinum* BSG101. Data is presented as box and whisker plots of activity scores. The solid line shown at 0 is the median control value while the dotted line at 1 is the activity threshold. Scores above 1 correspond to a >90% reduction in bacterial bioluminescence compared to the corresponding no-fungi control. Similarly, an activity score above 2 means corresponds to a >99% reduction. CSA, Czapek Solution Agar; CYA, Czapek Yeast Extract Agar; MEA, Malt Extract Agar; OA, Oatmeal Agar; PDA, Potato Dextrose Agar; TYA, Tryptone Yeast Extract Agar. Boxes are upper and lower quartiles with median shown. The whiskers extend up to 1.5× the inter-quartile range and any dots beyond those bounds are outliers.

**FIGURE 7:**
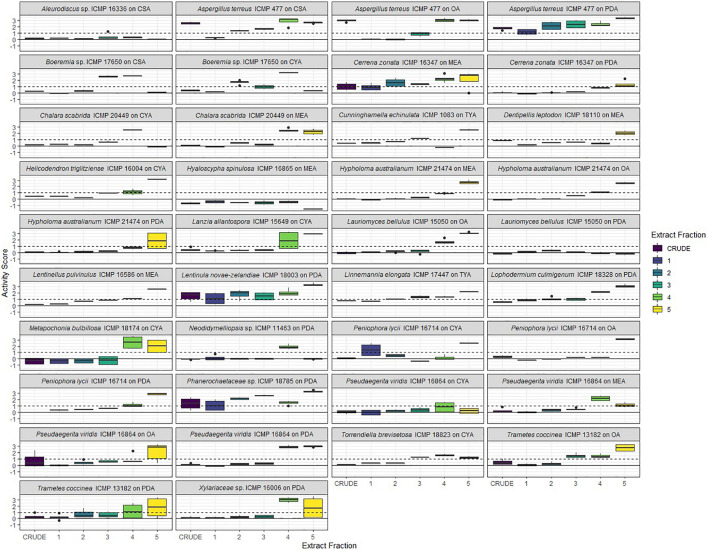
Antibacterial activity of crude extracts and fractions 1–5 from ICMP fungal isolates against *Mycobacterium abscessus* BSG301. Data is presented as box and whisker plots of activity scores. The solid line shown at 0 is the median control value while the dotted line at 1 is the activity threshold. Scores above 1 correspond to a >90% reduction in bacterial bioluminescence compared to the corresponding no-fungi control. Similarly, an activity score above 2 means corresponds to a >99% reduction. CSA, Czapek Solution Agar; CYA, Czapek Yeast Extract Agar; MEA, Malt Extract Agar; OA, Oatmeal Agar; PDA, Potato Dextrose Agar; TYA, Tryptone Yeast Extract Agar. Boxes are upper and lower quartiles with median shown. The whiskers extend up to 1.5× the inter-quartile range and any dots beyond those bounds are outliers.

We tested, at a single concentration of 1000 μg/mL, the crude extracts and fractions F1–F5 from all 41 fungus-medium combinations for activity against *M. marinum* BSG101 (Figures 5, 6) and for 38 of the combinations for activity against *M. abscessus* BSG301 (Figures 5, 7). As described previously, we measured antibacterial activity as reductions in light output of our bioluminescent mycobacterial strains over a 72-h period and calculated activity scores as the negative log of the ratio of the AUC values of the fungus-containing measurements and the control measurements. We define an extract/fraction as active/antibacterial if the median activity score is above 1, which corresponds to a >90% reduction in light compared to the control, as previously described. Similarly, an activity score above 2 means corresponds to a > 99% reduction.

#### ICMP Fungal Extracts and Fractions Retain Anti-mycobacterial Activity

We observed that only 3/38 of the fungus-medium combinations we tested for activity against *M. abscessus* BSG301 did not retain any activity in either the crude extract or any of the 5 fractions. These belonged to *Aleurodiscus* sp. ICMP 16336 grown on CSA, *Pseudaegerita viridis* ICMP 16864 grown on CYA, and *Lauriomyces bellulus* ICMP 15050 grown on PDA (Figures 5, 7). Of the remaining 14 Basidiomycota-medium combinations tested, the most active fraction was F5 [14/14 (100%)], followed by F4 [7/14 (50%)]. Four Basidiomycota-medium combinations also displayed some activity from fractions F2 and/or F3. Of the 17 Ascomycota-medium combinations we observed to be active, the most active fraction was F4 [17/17 (100%)], followed by F5 [13/17 (76%)]. Six Ascomycota-medium combinations also displayed some activity from fractions F2 and/or F3. Both Mucoromycota-medium combinations tested had multiple active fractions.

We observed that all 41 of the fungus-medium combinations we tested for activity against *M. marinum* BSG101 retained some activity in either the crude extract or at least one of the 5 fractions (Figures 5, 6). Of the 18 Basidiomycota-medium combinations tested, the most active fraction was F5 [17/18 (94%)], followed by F4 [11/17 (65%)]. Seven Basidiomycota-medium combinations also displayed some activity from fractions F2 and/or F3. Like the Basidiomycota-medium combinations, of the 21 Ascomycota-medium combinations we observed to be active, the most active fraction was F5 [21/21 (100%)], followed by F4 [16/21 (76%)]. Eight Ascomycota-medium combinations also displayed some activity from fractions F2 and/or F3. Both Mucoromycota-medium combinations tested had multiple active fractions.

#### Linoleic Acid Is Likely the Anti-mycobacterial Compound Present in Fraction F5

Given the anti-mycobacterial activity we observed from the F5 fractions of so many of the ICMP isolates, we analyzed this fraction in more detail from four phylogenetically diverse ICMP isolates: the Basidiomycota *Aleurodiscus* sp. ICMP 16336, the Ascomycota *Hyaloscypha spinulosa* ICMP 16865 and *Lanzia allantospora* ICMP 15649, and the Mucoromycota *Cunninghamella echinulata* ICMP 1083. NMR spectroscopic and Mass spectrometric analysis confirmed the presence of linoleic acid in fraction F5 of these fungi.

#### Identification of Active ICMP Fungal Fractions for Further Analysi*s*

To prioritize the most active anti-mycobacterial fungus-medium combinations for further NMR spectroscopic and Mass spectrometric analysis, we tested those fractions F2, F3, and F4 that were active at 1000 mg/mL to obtain their minimum inhibitory concentration (MIC) (Table 4).

**TABLE 4:** Minimum inhibitory concentrations (MIC) of ICMP fungal fractions (F2, F3, and F4) against *M. abscessus* and *M. marinum*.

Species	ICMP	Media^1^	*M. abscessus* MIC (μg/mL)			*M. marinum* MIC (μg/mL)		
				
			F2	F3	F4	F2	F3	F4
*Aspergillus terreus*	477	CSA	1000	**125**	1000	**250**	**500**	**125**
		OA	>1000	**250**	>1000	>1000	1000	**250**
		PDA	**500**	**500**	**500**	**500**	**500**	**500**
*Boeremia* sp.	17650	CSA	1000	**500**	>1000	>1000	>1000	1000
		CYA	>1000	**250**	1000	>1000	>1000	**500**
*Cerrena zonata*	16347	MEA	1000	1000	1000	1000	1000	**500**
		PDA	>1000	>1000	>1000	>1000	>1000	1000
*Chalara scabrida*	20449	CYA	>1000	1000	>1000	>1000	1000	>1000
		MEA	>1000	1000	>1000	>1000	>1000	**500**
*Cunninghamella echinulata*	1083	TYA	1000	>1000	>1000	1000	**500**	>1000
*Hypholoma australianum*	21474	MEA	>1000	>1000	>1000	>1000	>1000	**500**
		OA	>1000	1000	>1000	>1000	>1000	1000
		PDA	>1000	>1000	>1000	>1000	>1000	1000
*Lauriomyces bellulus*	15050	OA	>1000	1000	>1000	>1000	>1000	1000
		PDA	>1000	>1000	>1000	>1000	>1000	>1000
*Lentinellus pulvinulus*	16586	MEA	>1000	1000	>1000	>1000	>1000	1000
*Lentinula novae-zelandiae*	18003	PDA	1000	1000	1000	1000	**500**	**500**
*Linnemannia elongate*	17447	TYA	1000	1000	1000	1000	>1000	1000
*Lophodermium culmigenum*	18328	PDA	1000	**500**	>1000	1000	1000	**62.5**
*Metapochonia bulbillosa*	18174	CYA	>1000	**250**	1000	>1000	**500**	**500**
*Neodidymelliopsis* sp.	11463	PDA	>1000	1000	>1000	>1000	>1000	**500**
*Peniophora lycii*	16714	PDA	>1000	>1000	>1000	>1000	1000	1000
*Phanerochaetaceae* sp.	18785	PDA	**500**	1000	1000	**500**	**250**	1000
*Pseudaegerita viridis*	16864	CYA	>1000	>1000	>1000	>1000	>1000	**250**
		MEA	>1000	**500**	>1000	1000	>1000	**62.5**
		OA	>1000	>1000	>1000	>1000	>1000	**250**
		PDA	>1000	**31.25**	>1000	1000	>1000	**31.25**
*Torrendiella brevisetosa*	18823	CYA	**500**	**500**	>1000	>1000	1000	>1000
*Trametes coccinea*	13182	OA	1000	1000	>1000	>1000	1000	**500**
		PDA	>1000	1000	>1000	1000	1000	**250**
*Xylariaceae* sp.	16006	PDA	>1000	**500**	>1000	>1000	>1000	1000

*^1^CSA, Czapek Solution Agar; CYA, Czapek Yeast Extract Agar; MEA, Malt Extract Agar; OA, Oatmeal Agar; PDA, Potato Dextrose Agar; TYA, Tryptone Yeast Extract Agar. MIC values ≤500 μg/mL are shown in bold.*

Of the 31 fungus-medium combinations tested against *M. abscessus* BSG301, 11/31 (35%) F3 fractions had an MIC ≤ 500 μg/mL. Of these, the most active fungus-medium combinations were *Pseudaegerita viridis* ICMP 16864 grown on PDA with an MIC of 31.25 μg/mL, and *Aspergillus terreus* ICMP 477 grown on CSA, with an MIC of 125 μg/mL (Table 4). In contrast, only three F4 fractions and one F2 fraction had an MIC ≤ 500 μg/mL.

In contrast to *M. abscessus*, of the 31 fungus-medium combinations tested against *M. marinum* BSG101, the most active fractions were F4 rather than F3, with 17/31 (55%) F4 fractions having an MIC ≤ 500 μg/mL. Of these, the most active fungus-medium combinations were *Pseudaegerita viridis* ICMP 16864 grown on PDA and MEA, with an MIC of 31.25 and 62.5 μg/mL, respectively, and *Lophodermium culmigenum* ICMP 18328, with an MIC of 62.5 μg/mL (Table 4). Three F2 fractions and six F3 fractions had an MIC ≤ 500 μg/mL.

## Discussion

In this study, we describe a medium-throughput bioluminescence-based pipeline to screen fungi for activity against Mycobacteria using bioluminescent derivates of *M. abscessus* and *M. marinum* as the testing strains. We included *M. abscessus* as it is a relatively fast-growing non-tuberculous mycobacterial species and is a cause of opportunistic infections in patients with cystic fibrosis or chronic pulmonary disease, and of skin and soft tissue infections, for which treatment options are limited (To et al., 2020; Victoria et al., 2021). We also included *M. marinum* as, despite being a pathogen of fish, amphibians, and reptiles, it shares conserved virulence determinants with *M. tuberculosis* (Bouz and Al Hasawi, 2018; Ramakrishnan, 2020) and is a Biosafety Level (BSL) 2 rather than an airborne BSL 3 organism.

We screened 36 ICMP fungal isolates using our assay and discovered that almost all produced considerable anti-mycobacterial activity. This is in contrast with our experience screening ICMP isolates for activity against other human pathogens such as *Escherichia coli*, *Pseudomonas aeruginosa*, and *Staphylococcus aureus*, where we find just 5–20% of fungal isolates have some antibacterial activity (unpublished data). We also observed some differences between the two mycobacterial species, with more ICMP isolates being active against *M. marinum* than *M. abscessus*. This is not unsurprising given their different ecological niches and divergent genomes (Malhotra et al., 2017). Identification of the chemical compounds responsible may shed some further light on these differences.

Our screening pipeline involves growing fungi in 24 well plates on multiple growth media. It has previously been shown that different culture conditions can alter the expression of biosynthetic gene clusters and therefore the structural diversity and quantity of secondary metabolites produced by microorganisms, including fungi (Bills et al., 2008). For example, growing *Fusarium tricinctum* on Rice medium supplemented with fruit and vegetable juices led to the discovery of Fusarielin J (Hemphill et al., 2017) while growing *Asteromyces cruciatus* on Czapek-Dox medium with an altered nitrogen source led to the discovery of Lajollamide A (Gulder et al., 2012). We selected media that cover a broad range of carbon and nitrogen sources, as well as different pH and metal ions. We have also included media with less chemically defined elements, including from potatoes, rice, and oatmeal. Our data provides strong statistical evidence for the impact of growth media on antibacterial activity of the ICMP fungi we tested, with some fungal isolates only active when grown on one medium and others active when grown on several, or even all. The highest proportion of ICMP isolates were active when grown on the nutritionally rich Oatmeal Agar (OA), Potato Dextrose Agar (PDA), and (MYA) while the lowest proportion were active when grown on the more nutritionally poor Rice Extract Agar (REA) and Czapek Solution Agar (CSA).

The ICMP fungi we screened in this project included isolates of several species well known to produce antimicrobial compounds. For example, *Aspergillus terreus* produces terrein (Goutam et al., 2017), maunakeanolic acid A and B (Zaman et al., 2020), and helvolic acid (Zaman et al., 2020), amongst other compounds. Chemical extraction and fractionation of a subset of the ICMP isolates revealed that much of the activity we observed may be due to the production of the known anti-mycobacterial compound linoleic acid (Kanetsuna, 1985; Choi, 2016). However, we have identified several ICMP isolates that retained their anti-mycobacterial activity in non-linoleic acid containing fractions. These include isolates of *Lophodermium culmigenum*, *Pseudaegerita viridis*, and *Trametes coccinea*, as well as an unknown species of *Boeremia* and an isolate of an unknown genus and species in the family *Phanerochaetaceae*. Investigations are ongoing to identify the sources of their anti-mycobacterial activity and to determine whether any may be due to the production of novel bioactive compounds. Species of *Lophodermium* and *Pseudaegerita* have previously been found to produce several antifungal compounds (Hosoya et al., 2007; Sumarah et al., 2011; McMullin et al., 2015), while *T. coccinea* is predicted to have secondary metabolite pathways though genomic analysis (Zhang et al., 2020).

An interesting observation we have made, is of the abundant anti-mycobacterial activity of fungi we tested in the order Polyporales. The isolates *Cerrena zonata* ICMP 16347, *Laetiporus portentosus* ICMP 15555, *Phanerochaetaceae* sp. ICMP 18785, and *T. coccinea* ICMP 13182, were active against both mycobacterial species when grown in almost all media. These fungi are bracket-like fungi with pores on the under surface. To fulfill their ecological niche of digesting moist wood, these fungi first need to colonize the wood. To do this they need to compete with other microorganisms, including bacteria, and producing antimicrobial compounds would be beneficial on this process. An alternative hypothesis could be that their antibacterial activity is a by-product of these fungi producing the peroxidases and oxidases they need to digest wood (Sulej et al., 2019). Should the activity prove not to be the result of peroxidase/oxidase production, this would suggest that focusing future screening efforts on Polyporales fungi could prove fruitful for the discovery of new antibacterial compounds.

## Data Availability Statement

The datasets presented in this study can be found in online repositories. The names of the repository/repositories and accession number(s) can be found below: https://www.ncbi.nlm.nih.gov/genbank/, MT107903, MZ325955, MZ325952, MW862777, MW862790, MW862786, MK432752, MZ325951, MZ325956, MZ325966, MK432688, MK432695, MZ325972, MZ325953, AY755334, EF029218, MW862787, MZ325965, MZ325962, MZ325968, MZ325967, MZ325970, MZ325971, MW862783, MZ325959, MZ325969, MH395972, MH395959, GQ411512, MZ325960, MZ325961, JN225946, MW862784, EU770239, MZ325963, and MZ325954 and https://figshare.com/, https://auckland.figshare.com/articles/dataset/Antibacterial_activity_of_fungal_isolates_from_the_International_Collection_of_Microorganisms_from_Plants_ICMP_against_Mycobacterium_abscessus_and_Mycobacterium_marinum_/14937894.

## Author Contributions

BW, BC, and SW contributed to conception and design of the study. AG, MC, YD, and DP performed the experiments. AG, MC, TL, BW, and SW were involved in data analysis. AG and SW wrote the manuscript. All authors contributed to manuscript revision, read, and approved the submitted version.

## Conflict of Interest

The authors declare that the research was conducted in the absence of any commercial or financial relationships that could be construed as a potential conflict of interest.

## Publisher’s Note

All claims expressed in this article are solely those of the authors and do not necessarily represent those of their affiliated organizations, or those of the publisher, the editors and the reviewers. Any product that may be evaluated in this article, or claim that may be made by its manufacturer, is not guaranteed or endorsed by the publisher.
