# Supplementary material for: Screening of Fungi for Antimycobacterial Activity Using a Medium-Throughput Bioluminescence-Based Assay
Source: Front Microbiol. 2021 Sep 6;12:739995. doi: 10.3389/fmicb.2021.739995 (PMC8450596; doi:10.3389/fmicb.2021.739995)
Supplement: Supplementary file 1 [file Data_Sheet_1.docx]

Supplementary Material

# Supplementary Methods: Extraction and purification procedures

Cultures of *Agaricales* sp. (ICMP 17554) were grown on 39 PDA plates at room temperature and freeze-dried. The dry cultures (21.17 g, dry weight) were extracted with MeOH (2 x 1 L) for 4 hours followed CH_2_Cl_2_ (1 L) overnight. Concentration of the combined organic extracts under reduced pressure afforded a brown oil (6.51 g). The crude extract was subjected to C_8_ reversed-phase column chromatography eluting with gradient H_2_O/MeOH to afford five fractions (F1–F5).

Cultures of *Agaricales* sp. (ICMP 17554) were grown on 45 MEA plates at room temperature and freeze-dried. The dry cultures (45.92 g, dry weight) were extracted with MeOH (2 x 1 L) for 4 hours followed CH_2_Cl_2_ (1 L) overnight. Concentration of the combined organic extracts under reduced pressure afforded a brown oil (9.75 g). The crude extract was subjected to C_8_ reversed-phase column chromatography eluting with gradient H_2_O/MeOH to afford five fractions (F1–F5).

A 1 L liquid culture of *Agaricales* sp. (ICMP 17554) was grown in PDB at room temperature and filtered. The liquid culture was extracted with ethyl acetate (3 x 500 mL) and the combined organic extracts dried over anhydrous MgSO_4_ and solvent removed under reduced pressure to afford an orange oil (0.66 g). The crude extract was subjected to C_8_ reversed-phase column chromatography eluting with gradient H_2_O/MeOH to afford five fractions (F1–F5).

Cultures of *Aleurodiscus* sp. (ICMP 16336) were grown on 56 CSA plates at room temperature and freeze-dried. The dry cultures (49.02 g, dry weight) were extracted with MeOH (2 x 1.5 L) for 4 hours followed CH_2_Cl_2_ (1.5 L) overnight. Concentration of the combined organic extracts under reduced pressure afforded a brown oil (22.18 g). The crude extract was subjected to C_8_ reversed-phase column chromatography eluting with gradient H_2_O/MeOH to afford five fractions (F1–F5).

Cultures of *Aspergillus terreus* (ICMP 477) were grown on 40 PDA plates at room temperature and freeze-dried. The dry cultures (26.57 g, dry weight) were extracted with MeOH (2 x 1 L) for 4 hours followed CH_2_Cl_2_ (1 L) overnight. Concentration of the combined organic extracts under reduced pressure afforded a brown oil (2.45 g). The crude extract was subjected to C_8_ reversed-phase column chromatography eluting with gradient H_2_O/MeOH to afford five fractions (F1–F5).

Cultures of *Aspergillus terreus* (ICMP 477) were grown on 39 OEA plates at room temperature and freeze-dried. The dry cultures (36.82 g, dry weight) were extracted with MeOH (2 x 1 L) for 4 hours followed CH_2_Cl_2_ (1 L) overnight. Concentration of the combined organic extracts under reduced pressure afforded a brown oil (4.94 g). The crude extract was subjected to C_8_ reversed-phase column chromatography eluting with gradient H_2_O/MeOH to afford five fractions (F1–F5).

Cultures of *Aspergillus terreus* (ICMP 477) were grown on 40 CSA plates at room temperature and freeze-dried. The dry cultures (22.55 g, dry weight) were extracted with MeOH (2 x 700 mL) for 4 hours followed CH_2_Cl_2_ (700 mL) overnight. Concentration of the combined organic extracts under reduced pressure afforded a brown oil (2.98 g). The crude extract was subjected to C_8_ reversed-phase column chromatography eluting with gradient H_2_O/MeOH to afford five fractions (F1–F5).

Cultures of *Boeremia* sp. (ICMP 17650) were grown on 37 CYA plates at room temperature and freeze-dried. The dry cultures (28.68 g, dry weight) were extracted with MeOH (2 x 750 mL) for 4 hours followed CH_2_Cl_2_ (750 mL) overnight. Concentration of the combined organic extracts under reduced pressure afforded a brown oil (4.00 g). The crude extract was subjected to C_8_ reversed-phase column chromatography eluting with gradient H_2_O/MeOH to afford five fractions (F1–F5).

Cultures of *Boeremia* sp. (ICMP 17650) were grown on 39 CSA plates at room temperature and freeze-dried. The dry cultures (32.91 g, dry weight) were extracted with MeOH (2 x 750 mL) for 4 hours followed CH_2_Cl_2_ (750 mL) overnight. Concentration of the combined organic extracts under reduced pressure afforded a brown oil (12.97 g). The crude extract was subjected to C_8_ reversed-phase column chromatography eluting with gradient H_2_O/MeOH to afford five fractions (F1–F5).

A 1 L liquid culture of *Boeremia* sp. (ICMP 17650) was grown in PDB at room temperature and filtered. The liquid culture was extracted with ethyl acetate (3 x 500 mL) and the combined organic extracts dried over anhydrous MgSO_4_ and solvent removed under reduced pressure to afford a pale brown oil (0.30 g). The crude extract was subjected to C_8_ reversed-phase column chromatography eluting with gradient H_2_O/MeOH to afford five fractions (F1–F5).

Cultures of *Cerrena zonata* (ICMP 16347) were grown on 51 MEA plates at room temperature and freeze-dried. The dry cultures (67.33 g, dry weight) were extracted with MeOH (2 x 1 L) for 4 hours followed CH_2_Cl_2_ (1 L) overnight. Concentration of the combined organic extracts under reduced pressure afforded a brown oil (4.76 g). The crude extract was subjected to C_8_ reversed-phase column chromatography eluting with gradient H_2_O/MeOH to afford five fractions (F1–F5).

Cultures of *Cerrena zonata* (ICMP 16347) were grown on 19 CYA plates at room temperature and freeze-dried. The dry cultures (15.88 g, dry weight) were extracted with MeOH (2 x 400 mL) for 4 hours followed CH_2_Cl_2_ (400 mL) overnight. Concentration of the combined organic extracts under reduced pressure afforded a brown oil (2.80 g). The crude extract was subjected to C_8_ reversed-phase column chromatography eluting with gradient H_2_O/MeOH to afford five fractions (F1–F5).

Cultures of *Cerrena zonata* (ICMP 16347) were grown on 44 PDA plates at room temperature and freeze-dried. The dry cultures (32.55 g, dry weight) were extracted with MeOH (2 x 1 L) for 4 hours followed CH_2_Cl_2_ (1 L) overnight. Concentration of the combined organic extracts under reduced pressure afforded a brown oil (10.49 g). The crude extract was subjected to C_8_ reversed-phase column chromatography eluting with gradient H_2_O/MeOH to afford five fractions (F1–F5).

Cultures of *Chalara scabrida* (ICMP 20449) were grown on 36 CYA plates at room temperature and freeze-dried. The dry cultures (30.52 g, dry weight) were extracted with MeOH (2 x 1 L) for 4 hours followed CH_2_Cl_2_ (1 L) overnight. Concentration of the combined organic extracts under reduced pressure afforded a brown oil (2.34 g). The crude extract was subjected to C_8_ reversed-phase column chromatography eluting with gradient H_2_O/MeOH to afford five fractions (F1–F5).

Cultures of *Chalara scabrida* (ICMP 20449) were grown on 38 MEA plates at room temperature and freeze-dried. The dry cultures (19.74 g, dry weight) were extracted with MeOH (2 x 1 L) for 4 hours followed CH_2_Cl_2_ (1 L) overnight. Concentration of the combined organic extracts under reduced pressure afforded a brown oil (1.81 g). The crude extract was subjected to C_8_ reversed-phase column chromatography eluting with gradient H_2_O/MeOH to afford five fractions (F1–F5).

Cultures of *Cunninghamella echinulata* (ICMP 1083) were grown on 34 TYEA plates at room temperature and freeze-dried. The dry cultures (12.72 g, dry weight) were extracted with MeOH (2 x 1 L) for 4 hours followed CH_2_Cl_2_ (1 L) overnight. Concentration of the combined organic extracts under reduced pressure afforded a brown oil (1.35 g). The crude extract was subjected to C_8_ reversed-phase column chromatography eluting with gradient H_2_O/MeOH to afford five fractions (F1–F5).

Cultures of *Dentipellis leptodon* (ICMP 18110) were grown on 26 MEA plates at room temperature and freeze-dried. The dry cultures (15.36 g, dry weight) were extracted with MeOH (2 x 400 mL) for 4 hours followed CH_2_Cl_2_ (400 mL) overnight. Concentration of the combined organic extracts under reduced pressure afforded a brown oil (1.62 g). The crude extract was subjected to C_8_ reversed-phase column chromatography eluting with gradient H_2_O/MeOH to afford five fractions (F1–F5).

Cultures of *Helicodendron triglitziense* (ICMP 16004) were grown on 40 CYA plates at room temperature and freeze-dried. The dry cultures (47.09 g, dry weight) were extracted with MeOH (2 x 1 L) for 4 hours followed CH_2_Cl_2_ (1 L) overnight. Concentration of the combined organic extracts under reduced pressure afforded a brown oil (11.58 g). The crude extract was subjected to C_8_ reversed-phase column chromatography eluting with gradient H_2_O/MeOH to afford five fractions (F1–F5).

Cultures of *Hyaloscypha spinulosa* (ICMP 16865) were grown on 59 PDA plates at room temperature and freeze-dried. The dry cultures (70.67 g, dry weight) were extracted with MeOH (2 x 1 L) for 4 hours followed CH_2_Cl_2_ (1 L) overnight. Concentration of the combined organic extracts under reduced pressure afforded a brown oil (2.38 g). The crude extract was subjected to C_8_ reversed-phase column chromatography eluting with gradient H_2_O/MeOH to afford five fractions (F1–F5).

Cultures of *Hyaloscypha spinulosa* (ICMP 16865) were grown on 37 MEA plates at room temperature and freeze-dried. The dry cultures (18.44 g, dry weight) were extracted with MeOH (2 x 1 L) for 4 hours followed CH_2_Cl_2_ (1 L) overnight. Concentration of the combined organic extracts under reduced pressure afforded a brown oil (1.16 g). The crude extract was subjected to C_8_ reversed-phase column chromatography eluting with gradient H_2_O/MeOH to afford five fractions (F1–F5).

Cultures of *Hypholoma australianum* (ICMP 21474) were grown on 30 OEA plates at room temperature and freeze-dried. The dry cultures (45.50 g, dry weight) were extracted with MeOH (2 x 750 mL) for 4 hours followed CH_2_Cl_2_ (750 mL) overnight. Concentration of the combined organic extracts under reduced pressure afforded a brown oil (11.45 g). The crude extract was subjected to C_8_ reversed-phase column chromatography eluting with gradient H_2_O/MeOH to afford five fractions (F1–F5).

Cultures of *Hypholoma australianum* (ICMP 21474) were grown on 38 MEA plates at room temperature and freeze-dried. The dry cultures (22.18 g, dry weight) were extracted with MeOH (2 x 1 L) for 4 hours followed CH_2_Cl_2_ (1 L) overnight. Concentration of the combined organic extracts under reduced pressure afforded a brown oil (8.26 g). The crude extract was subjected to C_8_ reversed-phase column chromatography eluting with gradient H_2_O/MeOH to afford five fractions (F1–F5).

Cultures of *Hypholoma australianum* (ICMP 21474) were grown on 40 PDA plates at room temperature and freeze-dried. The dry cultures (33.22 g, dry weight) were extracted with MeOH (2 x 1 L) for 4 hours followed CH_2_Cl_2_ (1 L) overnight. Concentration of the combined organic extracts under reduced pressure afforded a brown oil (13.87 g). The crude extract was subjected to C_8_ reversed-phase column chromatography eluting with gradient H_2_O/MeOH to afford five fractions (F1–F5).

Cultures of *Lanzia allantospora* (ICMP 15649) were grown on 38 CYA plates at room temperature and freeze-dried. The dry cultures (42.98 g, dry weight) were extracted with MeOH (2 x 1 L) for 4 hours followed CH_2_Cl_2_ (1 L) overnight. Concentration of the combined organic extracts under reduced pressure afforded a brown oil (14.52 g). The crude extract was subjected to C_8_ reversed-phase column chromatography eluting with gradient H_2_O/MeOH to afford five fractions (F1–F5).

Cultures of *Lauriomyces bellulus* (ICMP 15050) were grown on 45 PDA plates at room temperature and freeze-dried. The dry cultures (39.47 g, dry weight) were extracted with MeOH (2 x 350 mL) for 4 hours followed CH_2_Cl_2_ (350 mL) overnight. Concentration of the combined organic extracts under reduced pressure afforded an orange oil (10.17 g). The crude extract was subjected to C_8_ reversed-phase column chromatography eluting with gradient H_2_O/MeOH to afford five fractions (F1–F5).

Cultures of *Lauriomyces bellulus* (ICMP 15050) were grown on 45 OEA plates at room temperature and freeze-dried. The dry cultures (58.52 g, dry weight) were extracted with MeOH (2 x 1 L) for 4 hours followed CH_2_Cl_2_ (1 L) overnight. Concentration of the combined organic extracts under reduced pressure afforded an orange oil (4.74 g). The crude extract was subjected to C_8_ reversed-phase column chromatography eluting with gradient H_2_O/MeOH to afford five fractions (F1–F5).

Cultures of *Lauriomyces bellulus* (ICMP 15050) were grown on 44 MEA plates at room temperature and freeze-dried. The dry cultures (35.74 g, dry weight) were extracted with MeOH (2 x 800 mL) for 4 hours followed CH_2_Cl_2_ (800 mL) overnight. Concentration of the combined organic extracts under reduced pressure afforded an orange oil (4.07 g). The crude extract was subjected to C_8_ reversed-phase column chromatography eluting with gradient H_2_O/MeOH to afford five fractions (F1–F5).

Cultures of *Lentinula novae-zelandiae* (ICMP 18003) were grown on 55 PDA plates at room temperature and freeze-dried. The dry cultures (51.06 g, dry weight) were extracted with MeOH (2 x 1 L) for 4 hours followed CH_2_Cl_2_ (1 L) overnight. Concentration of the combined organic extracts under reduced pressure afforded a brown oil (25.44 g). The crude extract was subjected to C_8_ reversed-phase column chromatography eluting with gradient H_2_O/MeOH to afford five fractions (F1–F5).

A 1 L liquid culture of *Lentinula novae-zelandiae* (ICMP 18003) was grown in PDB at room temperature and filtered. The liquid culture was extracted with ethyl acetate (3 x 500 mL) and the combined organic extracts dried over anhydrous MgSO_4_ and solvent removed under reduced pressure to afford an orange oil (0.60 g). The crude extract was subjected to C_8_ reversed-phase column chromatography eluting with gradient H_2_O/MeOH to afford five fractions (F1–F5).

Cultures of *Lophodermium culmigenum* (ICMP 18328) were grown on 59 PDA plates at room temperature and freeze-dried. The dry cultures (22.65 g, dry weight) were extracted with MeOH (2 x 750 mL) for 4 hours followed CH_2_Cl_2_ (750 mL) overnight. Concentration of the combined organic extracts under reduced pressure afforded a brown oil (0.77 g). The crude extract was subjected to C_8_ reversed-phase column chromatography eluting with gradient H_2_O/MeOH to afford five fractions (F1–F5).

Cultures of *Neodidymelliopsis* (ICMP 11463) were grown on 55 PDA plates at room temperature and freeze-dried. The dry cultures (108.6 g, dry weight) were extracted with MeOH (2 x 1 L) for 4 hours followed CH_2_Cl_2_ (1 L) overnight. Concentration of the combined organic extracts under reduced pressure afforded a red oil (0.51 g). The crude extract was subjected to C_8_ reversed-phase column chromatography eluting with gradient H_2_O/MeOH to afford five fractions (F1–F5).

Cultures of *Neodidymelliopsis* (ICMP 11463) were grown on 31 CYA plates at room temperature and freeze-dried. The dry cultures (17.22 g, dry weight) were extracted with MeOH (2 x 1 L) for 4 hours followed CH_2_Cl_2_ (1 L) overnight. Concentration of the combined organic extracts under reduced pressure afforded a red oil (0.59 g). The crude extract was subjected to C_8_ reversed-phase column chromatography eluting with gradient H_2_O/MeOH to afford five fractions (F1–F5).

A 1 L liquid culture of *Neodidymelliopsis* (ICMP 11463) was grown in PDB at room temperature and filtered. The liquid culture was extracted with ethyl acetate (3 x 500 mL) and the combined organic extracts dried over anhydrous MgSO_4_ and solvent removed under reduced pressure to afford an orange oil (0.66 g). The crude extract was subjected to C_8_ reversed-phase column chromatography eluting with gradient H_2_O/MeOH to afford five fractions (F1–F5).

Cultures of *Peniophora lycii* (ICMP 16714) were grown on 40 PDA plates at room temperature and freeze-dried. The dry cultures (22.17 g, dry weight) were extracted with MeOH (2 x 1 L) for 4 hours followed CH_2_Cl_2_ (1 L) overnight. Concentration of the combined organic extracts under reduced pressure afforded a brown oil (3.30 g). The crude extract was subjected to C_8_ reversed-phase column chromatography eluting with gradient H_2_O/MeOH to afford five fractions (F1–F5).

Cultures of *Peniophora lycii* (ICMP 16714) were grown on 37 OEA plates at room temperature and freeze-dried. The dry cultures (36.12 g, dry weight) were extracted with MeOH (2 x 700 mL) for 4 hours followed CH_2_Cl_2_ (700 mL) overnight. Concentration of the combined organic extracts under reduced pressure afforded a brown oil (3.88 g). The crude extract was subjected to C_8_ reversed-phase column chromatography eluting with gradient H_2_O/MeOH to afford five fractions (F1–F5).

Cultures of *Peniophora lycii* (ICMP 16714) were grown on 40 CYA plates at room temperature and freeze-dried. The dry cultures (29.02 g, dry weight) were extracted with MeOH (2 x 800 mL) for 4 hours followed CH_2_Cl_2_ (800 mL) overnight. Concentration of the combined organic extracts under reduced pressure afforded a brown oil (7.70 g). The crude extract was subjected to C_8_ reversed-phase column chromatography eluting with gradient H_2_O/MeOH to afford five fractions (F1–F5).

Cultures of *Peniophora lycii* (ICMP 16714) were grown on 71 MYA plates at room temperature and freeze-dried. The dry cultures (27.83 g, dry weight) were extracted with MeOH (2 x 1.5 L) for 4 hours followed CH_2_Cl_2_ (1.5 L) overnight. Concentration of the combined organic extracts under reduced pressure afforded a brown oil (2.38 g). The crude extract was subjected to C_8_ reversed-phase column chromatography eluting with gradient H_2_O/MeOH to afford five fractions (F1–F5).

Cultures of *Phanerochaetaceae* sp. (ICMP 18785) were grown on 35 PDA plates at room temperature and freeze-dried. The dry cultures (21.12 g, dry weight) were extracted with MeOH (2 x 1 L) for 4 hours followed CH_2_Cl_2_ (1 L) overnight. Concentration of the combined organic extracts under reduced pressure afforded a brown oil (4.51 g). The crude extract was subjected to C_8_ reversed-phase column chromatography eluting with gradient H_2_O/MeOH to afford five fractions (F1–F5).

Cultures of *Pseudaegerita viridis* (ICMP 16864) were grown on 41 PDA plates at room temperature and freeze-dried. The dry cultures (27.78 g, dry weight) were extracted with MeOH (2 x 750 mL) for 4 hours followed CH_2_Cl_2_ (750 mL) overnight. Concentration of the combined organic extracts under reduced pressure afforded a brown oil (2.58 g). The crude extract was subjected to C_8_ reversed-phase column chromatography eluting with gradient H_2_O/MeOH to afford five fractions (F1–F5).

Cultures of *Pseudaegerita viridis* (ICMP 16864) were grown on 40 MEA plates at room temperature and freeze-dried. The dry cultures (18.77 g, dry weight) were extracted with MeOH (2 x 500 mL) for 4 hours followed CH_2_Cl_2_ (500 mL) overnight. Concentration of the combined organic extracts under reduced pressure afforded a brown oil (1.87 g). The crude extract was subjected to C_8_ reversed-phase column chromatography eluting with gradient H_2_O/MeOH to afford five fractions (F1–F5).

Cultures of *Pseudaegerita viridis* (ICMP 16864) were grown on 40 OEA plates at room temperature and freeze-dried. The dry cultures (41.57 g, dry weight) were extracted with MeOH (2 x 750 mL) for 4 hours followed CH_2_Cl_2_ (750 mL) overnight. Concentration of the combined organic extracts under reduced pressure afforded a brown oil (6.03 g). The crude extract was subjected to C_8_ reversed-phase column chromatography eluting with gradient H_2_O/MeOH to afford five fractions (F1–F5).

Cultures of *Pseudaegerita viridis* (ICMP 16864) were grown on 42 CYA plates at room temperature and freeze-dried. The dry cultures (55.22 g, dry weight) were extracted with MeOH (2 x 1 L) for 4 hours followed CH_2_Cl_2_ (1 L) overnight. Concentration of the combined organic extracts under reduced pressure afforded a brown oil (21.53 g). The crude extract was subjected to C_8_ reversed-phase column chromatography eluting with gradient H_2_O/MeOH to afford five fractions (F1–F5).

Cultures of *Torrendiella brevisetosa* (ICMP 18823) were grown on 57 CYA plates at room temperature and freeze-dried. The dry cultures (117.21 g, dry weight) were extracted with MeOH (2 x 1 L) for 4 hours followed CH_2_Cl_2_ (1 L) overnight. Concentration of the combined organic extracts under reduced pressure afforded a brown oil (18.93 g). The crude extract was subjected to C_8_ reversed-phase column chromatography eluting with gradient H_2_O/MeOH to afford five fractions (F1–F5).

Cultures of *Trametes coccinea* (ICMP 13182) were grown on 50 PDA plates at room temperature and freeze-dried. The dry cultures (25.36 g, dry weight) were extracted with MeOH (2 x 1 L) for 4 hours followed CH_2_Cl_2_ (1 L) overnight. Concentration of the combined organic extracts under reduced pressure afforded a brown oil (1.10 g). The crude extract was subjected to C_8_ reversed-phase column chromatography eluting with gradient H_2_O/MeOH to afford five fractions (F1–F5).

Cultures of *Trametes coccinea* (ICMP 13182) were grown on 20 OEA plates at room temperature and freeze-dried. The dry cultures (29.88 g, dry weight) were extracted with MeOH (2 x 400 mL) for 4 hours followed CH_2_Cl_2_ (400 mL) overnight. Concentration of the combined organic extracts under reduced pressure afforded a brown oil (2.70 g). The crude extract was subjected to C_8_ reversed-phase column chromatography eluting with gradient H_2_O/MeOH to afford five fractions (F1–F5).

Cultures of *Xylariaceae* sp. (ICMP 16006) were grown on 36 PDA plates at room temperature and freeze-dried. The dry cultures (25.25 g, dry weight) were extracted with MeOH (2 x 650 mL) for 4 hours followed CH_2_Cl_2_ (650 mL) overnight. Concentration of the combined organic extracts under reduced pressure afforded a brown oil (3.24 g). The crude extract was subjected to C_8_ reversed-phase column chromatography eluting with gradient H_2_O/MeOH to afford five fractions (F1–F5).

Cultures of *Xylariaceae* sp. (ICMP 16006) were grown on 43 CEA plates at room temperature and freeze-dried. The dry cultures (35.54 g, dry weight) were extracted with MeOH (2 x 750 mL) for 4 hours followed CH_2_Cl_2_ (750 mL) overnight. Concentration of the combined organic extracts under reduced pressure afforded a brown oil (13.93 g). The crude extract was subjected to C_8_ reversed-phase column chromatography eluting with gradient H_2_O/MeOH to afford five fractions (F1–F5).
